# The Biphasic Effect of Flavonoids on Oxidative Stress and Cell Proliferation in Breast Cancer Cells

**DOI:** 10.3390/antiox11040622

**Published:** 2022-03-24

**Authors:** Xiaomin Xi, Jiting Wang, Yue Qin, Yilin You, Weidong Huang, Jicheng Zhan

**Affiliations:** College of Food Science and Nutritional Engineering, China Agricultural University, Beijing 100083, China; xxm1113@cau.edu.cn (X.X.); wangjiting@cau.edu.cn (J.W.); jnqinyue@cau.edu.cn (Y.Q.); yilinyou@cau.edu.cn (Y.Y.); weidonghuang@cau.edu.cn (W.H.)

**Keywords:** breast cancer, flavonoids, oxidative stress, inflammation, transcriptome, BRCA1

## Abstract

Flavonoids have been reported to play an essential role in modulating processes of cellular redox homeostasis such as scavenging ROS. Meanwhile, they also induce oxidative stress that exerts potent antitumor bioactivity. However, the contradiction between these two aspects still remains unclear. In this study, four typical flavonoids were selected and studied. The results showed that low-dose flavonoids slightly promoted the proliferation of breast cancer cells under normal growth via gradually reducing accumulated oxidative products and demonstrated a synergistic effect with reductants NAC or VC. Besides, low-dose flavonoids significantly reduced the content of ROS and MDA induced by LPS or Rosup but restored the activity of SOD. However, high-dose flavonoids markedly triggered the cell death via oxidative stress as evidenced by upregulated ROS, MDA and downregulated SOD activity that could be partly rescued by NAC pretreatment, which was also confirmed by antioxidative gene expression levels. The underlying mechanism of such induced cell death was pinpointed as apoptosis, cell cycle arrest, accumulated mitochondrial superoxide, impaired mitochondrial function and decreased ATP synthesis. Transcriptomic analysis of apigenin and quercetin uncovered that high-dose flavonoids activated TNF-α signaling, as verified through detecting inflammatory gene levels in breast cancer cells and RAW 264.7 macrophages. Moreover, we identified that BRCA1 overexpression effectively attenuated such oxidative stress, inflammation and inhibited ATP synthesis induced by LPS or high dose of flavonoids possibly through repairing DNA damage, revealing an indispensable biological function of BRCA1 in resisting oxidative damage and inflammatory stimulation caused by exogenous factors.

## 1. Introduction

Breast cancer remains the second most common cause of cancer mortality in women worldwide, although many advances in early detection and treatment have been explored. Breast cancer is categorized into three major subtypes based on the presence or absence of molecular biomarkers, namely estrogen or progesterone receptors and human epidermal growth factor 2 (ERBB2, formerly HER2): hormone-receptor-positive/ERBB2-negative (70% of patients), ERBB2-positive (15–20%) and triple-negative (TNBC, tumors lack the above standard molecular markers, 5%) [1]. To date, treatment options for breast cancer mainly include surgical resection, radiotherapy, chemotherapy and endocrine therapy based on hormone receptors. In recent years, immunotherapy represented by PD-1/PD-L1 has also been applied in breast cancer patients [2]. Unluckily, for people presenting metastatic disease, chemotherapy is the only strategy to select, with goals of prolonging life and palliating symptoms. However, most nonmetastatic breast cancer patients gradually obtained drug resistance in the later stage and suffered from great pain during long-term treatment [3]. Therefore, exploring novel therapies that are effective while minimizing side effects is essential in curtailing breast cancer.

Flavonoids, belonging to secondary metabolites broadly distributed in plants, have multiple biological and pharmacological activities in improving body health, emerging as promising options for breast cancer prevention and treatment. These compounds act as free radical scavengers and antioxidants, exhibiting anti-mutagenic, anti-inflammatory and antiviral effects [4]. Moreover, flavonoids have the ability to reduce plasma levels of low-density lipoproteins, inhibit platelet aggregation and reduce cell proliferation. The health benefits of flavonoids have been historically ascribed to their chelating and antioxidant properties led by their innate chemical structure. The presence of multiple hydroxy groups along with a highly conjugated delocalized electron system enhances the free radical scavenging nature of the flavonoids and its interference with the redox activity of the cell [5]. The excellent antioxidant properties of flavonoids are especially evident under the stimulation of external oxidation conditions, such as in protecting skin cells under ultraviolet radiation; alleviating the oxidation products produced by certain chronic diseases such as diabetes and obesity; and maintaining the balanced redox situation in brain neurons, especially in degenerative brain diseases [6,7,8,9,10,11]. In terms of cancer biology, a previous study found that apigenin and luteolin induced apoptosis and cell cycle arrest in MCF-7 and TNBC cell lines via downregulating PI3K/Akt and upregulating FOXO3a, which correlated with reactive oxygen species (ROS) removal [12]. Besides, quercetin and resveratrol widely existed in wine-inhibited MCF-7 cell line proliferation through neutralizing hydrogen peroxide (H_2_O_2_) [13]. Butein was shown to induce apoptosis, as evidenced by increased caspase-3 and caspase-9 activities; notably, it reduced the ROS levels with the dose of 10 µg/mL butein for 5 min, which was also confirmed in BT-474 xenografts [14]. Wu et al. proved Ziziphora clinopodioides flavonoids protected against H_2_O_2_-induced injury in HUVEC cells and this effect was related to the enhancement of antioxidant capacity and suppression of angiogenesis and apoptosis [15].

Nevertheless, flavonoids were noticed to induce ROS accumulation causing programmed cell death (e.g., apoptosis) or directly lead to severe necrosis, indicating extra innovative mechanisms accounting for such cell death via interfering with the redox balance. Apigenin was found to induce apoptosis in mouse macrophage ANA-1 cells through the increased ROS accumulation and high caspase-3 activity followed by activation of the mitogen-activated protein kinase (MAPK) pathway [16]. The flavanone naringenin induced ROS-dependent apoptosis in MDA-MB-468 cells, and myricetin presented inhibition of the growth of MDA-MB-231 and MDA-MB-468 cell lines in a pro-oxidative way [17,18,19]. Moreover, the flavone 5,7-dihydroxy-8-nitrochrysin dephosphorylated Akt signals and resulted in ROS-dependent cytotoxicity in HER2-positive MDA-MB-453 breast cancer cells [20,21]. The flavonoid silibinin also exerted strong apoptosis through ROS-dependent Notch-1/ERK/Akt pathway in MDA-MB-231 cells. In vivo studies also supported such a conclusion [22]. The myricetin derived from flavonoid oncamex triggered increased superoxide production and cytotoxicity in MDA-MB-231 xenografts [23]. In addition to the function of single-component flavonoids in inducing oxidative stress, extracts derived from natural products that contained mixtures of flavonoids also exerted similar effects, which will not be repeated here [24,25,26,27]. Although the above results demonstrated that flavonoids promoted cancer cell death by inducing oxidative stress at the phenotypical level, they did not explain further which factor of flavonoids determined such quite opposite physiological activities on breast cancer cells. Moreover, these experiments were carried out with a single different drug, and the dosage, cancer cell line and culture conditions were quite different, making it difficult to obtain consistent results. Overall, the effect of flavonoids on the alteration of ROS level in breast cancer cells is still unclear. Therefore, in our study, we aimed to determine whether and how the different doses of flavonoids influenced the cellular oxidative stress and cell number with multiple flavonoids in breast cancer cells and tried to determine the underlying mechanism. In our current study, with four different kinds of flavonoids, we expound that a high dose of flavonoids (more than 20 μM) disturbed the cellular redox homeostasis, as indicated by extensive oxidative stress, and destroyed cellular ATP synthesis due to the impaired mitochondrial function, while a low dose of flavonoid (no more than 10 μM) pretreatment could markedly reverse LPS- or Rosup-induced oxidative stress via acting as an ROS scavenger.

## 2. Materials and Methods

### 2.1. Reagents and Chemicals

Lipopolysaccharides (LPSs) from *E. coli* 055:B5 were purchased from Solarbio (Cat. No. L8880, Beijing, China) with a purity > 99%. *N*-Acetyl-l-cysteine (NAC) and vitamin C (VC) were purchased from Beyotime (Cat. No. ST1546; Cat. No. ST1434, Shanghai, China) with a purity > 99%. The standard samples of apigenin, quercetin, chrysin and diosmetin were purchased from CHENGDU MUST Biotechnology CO.,LTD (Cat. No. A0113, A0083, A0292, A0927, MUST, Chengdu, China) with HPLC purity > 99%. Rabbit BRCA1 Polyclonal Antibody was purchased from Beyotime (Cat. No. AF6339, Shanghai, China). BeyoClick EdU 488 kit was purchased from Beyotime (Cat. No. C0071S, Shanghai, China).

### 2.2. Cell Culture

Human breast cancer cell lines MCF-7 and MDA-MB-231 and macrophage RAW 264.7 cells were cultured in DMEM (Gibco, Invitrogen, Carlsbad, CA, USA) with 10% fetal bovine serum (FBS; Gibco, Invitrogen, Carlsbad, CA, USA) and penicillin (100 U/mL)-streptomycin (100 mg/mL). Murine breast cancer 4T1 was cultured in RPMI-1640 (Gibco, Invitrogen, Carlsbad, CA, USA) with 10% fetal bovine serum, 100 U/mL penicillin, and 100 µg/mL streptomycin. Cells were all cultured in an incubator at 37 °C with 5% CO_2_ under sterile conditions. All cells were harvested by treatment with 0.25% trypsin–ethylenediaminetetraacetic acid (Trypsin-EDTA; Gibco, Invitrogen, Carlsbad, CA, USA). The information of above cell lines was listed in Supplementary Table S3.

### 2.3. Cell Number Detection and Colony Formation Assay

The cell counting kit-8 (CCK-8) assay (Cat. No. C0038, Beyotime, Shanghai, China) was used to detect cell numbers as measured by OD 450. Briefly, the cells were seeded in 100 μL of complete medium at a concentration of 3000-4000 cells per well in a 96-well plate overnight and then treated with indicated drugs and conditions. Then, according to the CCK-8 manufacturer’s instructions, a microplate reader (Thermo Fisher, Waltham, MA, USA) was used for the absorbance detection at a wavelength of 450 nm as OD value. For colony formation assay, 500-1000 cells were seeded in a 24-well plate overnight, treated with indicated drugs the next day and then cultured for 1–2 weeks. The cells were fixed with 4% paraformaldehyde (Cat. No. P1110, Solarbio, Beijing, China) for 15 min; then, they washed with PBS twice and stained with 1% crystal violet solution (Cat. No. C0121, Beyotime, Shanghai, China) for 10 min. Finally, the number of colonies was counted.

### 2.4. Detection of ROS, MDA and SOD

The content of reactive oxygen species (ROS) was detected by both fluorescence microplate reader (Sunrise/Infinite F50, Tecan, Mannedorf, Switzerland) and flow cytometry with DCFH-DA Assay (Cat. No. S0033M, Beyotime, Shanghai, China) according to protocol. The activity of superoxide dismutase (SOD) was detected by a commercial kit (Cat. No. S0101M, Beyotime, China). The concentration of malondialdehyde (MDA) was detected by a commercial kit (Cat. No. S0131S, Beyotime, Shanghai, China). All samples were prepared according to the introductions of each kit, and the OD values were assayed by a full wavelength microplate photometer (Thermo Fisher, Waltham, MA, USA).

### 2.5. Flow Cytometry for Apoptosis and Cell cycle

Apoptotic cells were assessed using FITC Annexin V Apoptosis Detection kit (Cat. No. C1062M, Beyotime, Shanghai, China), and cell cycle was detected by Cell Cycle Analysis Kit with propidium staining (Cat. No. C1052, Beyotime, Shanghai, China) according to protocol.

### 2.6. Real-Time Quantitative PCR Assay

Total RNA was extracted using TRIzol reagent (Cat. No. 9108, Takara Biotechnology, Dalian, China) from cells according to the manufacturer’s instructions, and then the RNA purity and concentration at 260:280 nm was measured. After diluting RNAs to the same concentration with RNase-free water, we used HiScript III 1st Strand cDNA Synthesis Kit (Cat. No. R312, Vazyme, Nanjing, China) to reversely transcribe 1 μg total RNAs and synthesize the first-strand cDNA according to the instructions. Real-time quantitative PCR was performed using ChamQ SYBR qPCR Master Mix (Cat. No. Q341, Vazyme, Nanjing, China). All analyses were performed via CFX Connect Real-Time System (Bio-Rad, Hercules, CA, USA). GAPDH was selected as the housekeeping gene to normalize the data of each target gene. The reaction conditions were as follows: 95 °C for 30 s, followed by 35 cycles of 95 °C for 5 s, 60 °C for 30 s, and melt curve. Results were presented as the fold change relative to the control. Three independent experiments were performed. All primers were listed in Supplementary Table S1.

### 2.7. Western Blotting

Cells were washed with cold PBS twice, and the total protein of cells was extracted using RIPA Lysis buffer (Cat. No. P0013B, Beyotime, Shanghai, China) premixed with 1 mM proteinase inhibitor PMSF. The protein concentration of cell lysis was detected using a BCA protein assay kit (Cat. No. P0012S, Beyotime, Shanghai, China). Then, samples were diluted to the same concentration and denatured with 5× SDS loading buffer (Cat. No. P0280, Beyotime, Shanghai, China). Protein was subjected to 10% SDS-PAGE by electrophoresis and transferred onto a polyvinylidene fluoride (PVDF) membrane. Membranes were incubated in 5% skimmed milk for 2 h at room temperature, followed by incubation with primary antibodies at 4 ℃ overnight. After being washed with 1× Tris-buffered saline containing Tween 20 (Cat. No. ST673, Beyotime, Shanghai, China), membranes were incubated with horseradish peroxidase (HRP)-conjugated secondary antibodies. Bands on the membrane were visualized by using BeyoECL Moon (Cat. No. P0018FS, Beyotime, Shanghai, China). For proteins of interest, band intensities were normalized to the housekeeping protein Histone H3. The detailed information of antibodies was listed in Supplementary Table S2.

### 2.8. Determination of Mitochondrial Membrane Potential

Mitochondrial membrane potential was determined in accordance with the standard procedures by mitochondrial membrane potential assay kit with JC-1 staining (Cat. No. C2006, Beyotime, Shanghai, China).

### 2.9. RNA-Seq Analysis

MCF-7 cells were treated with DMSO, 50 μM apigenin or quercetin for 48 h. Then, the total RNA was extracted and isolated with Oligo Magnetic Beads and randomly interrupted using divalent cations in NEB Fragmentation Buffer for cDNA synthesis. Libraries were generated using the NEB Next Ultra RNA Library Prep Kit for Illumina (New England BioLabs, Ipswich, MA, USA) following the manufacturer’s instructions. Sequencing was conducted using the Illumina NovaSeq 6000 platform.

### 2.10. Analysis of Enrichment Pathways

For Gene Ontology (GO) analysis, we used the online website tool (https://david.ncifcrf.gov/home.jsp, accessed on 1 November 2019) and then use R studio for visualization. For volcano plot and gene set enrichment analysis (GSEA) analysis, R studio software was used to analyze the data. The selection of differentially expressed genes from the transcriptome was based on the following standard: log_2_|fold change| > 1, *p* value < 0.01.

### 2.11. Quantitative ATP Detection

The ATP detection kit (Cat. No. S0026, Beyotime, Shanghai, China) was used to detect the concentration of ATP according to the manufacturer’s instructions. Briefly, cells were seeded in 2 mL of complete culture medium at the same number of 1 × 10^5^ cells per well in a six-well plate overnight, and then treated with indicated drugs according to the indicated design. The culture medium was removed, the cells were washed twice with cold PBS twice and the cells were lysed with 200 μL lysate per well. In order to fully lyse the cells, the cells were pipetted or the plate was shaken repeatedly to make the lysate completely contact the cells. Usually, cells could be lysed immediately when exposed to the lysate. The supernatant was obtained for subsequent determination via centrifuging at 4 ℃ and 12,000× *g* for 10 min. Standard curve and ATP detection working buffer were previously prepared according to the protocol. First, 100 μL ATP detection working buffer was added into the test holes of a 96-well plate, which was placed at room temperature for 5 min so that the background ATP was consumed. Then, 20 μL sample or standard was added into the test hole, which was quickly mixed with a micropipette, and the RLU value was measured with a luminometer after an interval of at least two seconds.

### 2.12. The Construction of BRCA1-Overexpressing Cells

The BRCA1 gene was subcloned from the cDNA of MCF-7 cells and inserted into the pLenti-CMV-Puro plasmid (Addgene, Watertown, MA, USA) and then verified by Sanger sequencing. HEK 293T cells were used to produce lentivirus with Lipo8000 Transfection Reagent (Cat. No. C0533, Beyotime, Shanghai, China). After cancer cells were seeded and cultured overnight, the fresh medium containing 5 μg/mL polybrene (Cat. No. ST1380, Beyotime, Shanghai, China) together with filtered lentivirus was used to culture cells for 24 h. Then, the cells were selected by puromycin (Cat. No. ST551, Beyotime, Shanghai, China) at an indicated concentration for a week to retain the successfully infected cells.

### 2.13. Flow Cytometry for CellROX Deep Red Probes

CellROX Deep Red probes (Cat. No. C10491, Thermo Fisher, Waltham, MA, USA) were used to detect the cellular oxidative stress by flow cytometry. Briefly, cells were seeded overnight and treated with indicated drugs the next day; then, they were collected and stained with probes according to protocol.

### 2.14. Mitochondrial Superoxide Staining

MitoSOX Red Indicator (Cat. No. 40778ES50, YEASEN, Shanghai, China) was applied to detect the cellular mitochondrial superoxide. Briefly, cells were seeded overnight and treated with indicated drugs the next day; then, they were stained with MitoSOX Red Indicator according to protocol. Then, cells were fixed with 4% paraformaldehyde and stained with DAPI (Cat. No. C1005, Beyotime, China) and then observed by confocal laser microscopy (Nikon, Tokyo, Japan).

### 2.15. DNA Damage Biomarker γ-H2AX Foci Detection

Briefly, cells treated with indicated flavonoid were washed with PBS and fixed for 10 min at room temperature with 4% paraformaldehyde (Cat. No. P0099, Beyotime, China) then permeabilized for 20 min on ice with 0.25% triton-X 100. Then, cells were washed twice with PBS and blocked with blocking buffer for 1 h at room temperature. Next, cells were incubated with the rabbit monoclonal antibody against phospho-histone H2AX (serine 139) (γ-H2AX, Cat. No. AF5836, Beyotime, China) at a 1:500 dilution for 1 h at room temperature. Cells were then washed twice with PBS and incubated for 1 h at room temperature with the secondary antibody Alexa Fluor 488 tagged anti-rabbit IgG (Cat. No. A0423, Beyotime, China). Cells were then washed twice and stained for 15 min with DAPI (Cat. No. C1005, Beyotime, China). Cells were then twice with PBS, and the slide was sealed. Fluorescence was observed by confocal laser microscopy (Nikon, Tokyo, Japan).

### 2.16. Statistical Analysis

All experiments were repeated three times independently with triplicates in each treatment. All data in this study were analyzed by unpaired Student’s *t*-test or one-way ANOVA (GraphPad Prism 8.0, La Jolla, CA, USA). Results are presented as the mean and standard error of the mean (mean ± SD). * *p* < 0.05, ** *p* < 0.01, *** *p* < 0.001, **** *p* < 0.0001. ns indicates no significant difference.

## 3. Results

### 3.1. Low-Dose Flavonoids Slightly Promoted the Growth of Breast Cancer Cells by Scavenging Accumulated Oxidative Products

**Figure 1:**
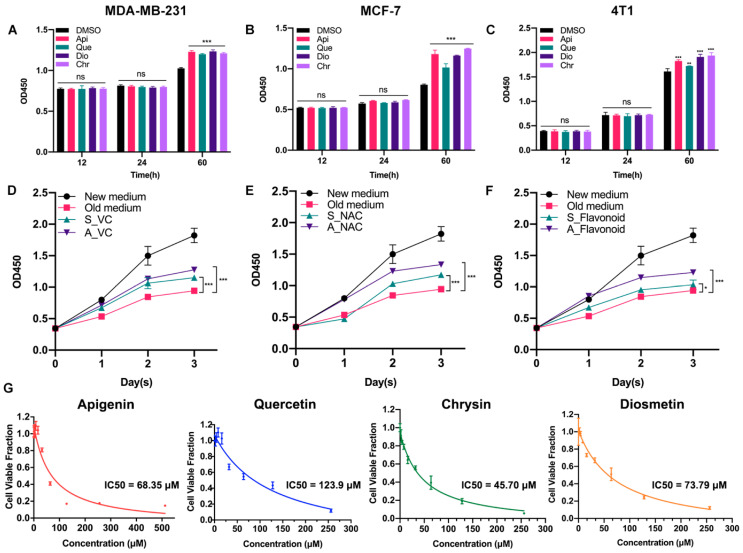
Low-dose flavonoids promoted cell growth by reducing oxidative reactions under long-term treatment. (**A**) The cell number of MDA-MB-231 treated with DMSO or flavonoids under 12, 24 or 60 h was detected. (**B**) The cell number of MCF-7 treated with DMSO or flavonoids under 12, 24 or 60 h was detected. (**C**) The cell number of 4T1 treated with DMSO or flavonoids under 12, 24 or 60 h was detected. (**D**) The growth rate of four groups. New medium: culturing the new seeded cells with fresh medium; Old medium: culturing the new seeded cells with old medium that had already cultured MCF-7 cells for 48 h. A_VC: culturing the new seeded cells with the above old medium but with VC supplement. S_VC: culturing the new seeded cells with collected medium in which VC had been simultaneously added for 48 h cotreatment in MCF-7 cells. VC: 500 μM. (**E**) The growth rate of four groups. The treatment was similar to (**D**). NAC: 5 μM. (**F**) The growth rate of four groups. The treatment was similar to (**D**). Flavonoid: apigenin, 5 μM. (**G**) The IC_50_ of four flavonoids was detected in MCF-7 cells. Data was shown as the mean ± SD. * *p* < 0.05, ** *p* < 0.01, *** *p* < 0.001. ns indicates no significant difference.

Previously, we found that low-dose apigenin (5 μM) could promote breast cancer cell growth. Therefore, four flavonoids with similar chemical structures were detected. Among the cell lines, MCF-7 and MDA-MB-231 belong to human beings and 4T1 comes from mice. Compared with the DMSO group, 5 μM flavonoids for 60 h, but not 12 or 24 h treatment, stimulated cell proliferation in all cell lines, suggesting that the growth promotion induced by low-dose flavonoids requires a relative long treating time (Figure 1A–C). We hypothesized that such a phenomenon was correlated with the accumulated oxidative products such as ROS during the long-period growth. Hence, we obtained the old medium by culturing MCF-7 cells for two days. Then, we added the old medium into the new seeded MCF-7 cells (old medium). The above old medium supplemented with VC (500 μM) was added into the new MCF-7 seeded cells altogether (A_VC). We also prepared another medium by simultaneously adding VC (500 μM) to the medium for 48 h cotreatment in MCF-7 cells, which was subsequently collected and added into the new seeded MCF-7 cells (S_VC). As shown in Figure 1D,E, the growth rate of A_VC was significantly higher than that of S_VC, indicating that the reductants were metabolized during normal cell growth and the post-addition of reductant was better. These results were in line with NAC (5 μM) groups. Moreover, low-dose flavonoids (apigenin, 5 μM) also displayed a similar function of acting as a reductant by alleviating the harmful oxidants (Figure 1F). To determine the cellular toxicity of four flavonoids on cancer cells, the IC_50_ was detected. Generally, chrysin showed the lowest IC_50_ with the strongest inhibitory effect in MCF-7 cells, followed by apigenin, diosmetin and quercetin, which was basically higher than 50 μM (Figure 1G).

### 3.2. Different Pharmacological Doses of Flavonoids Determined the Biphasic Biological Function in MCF-7 Breast Cancer Cells

**Figure 2:**
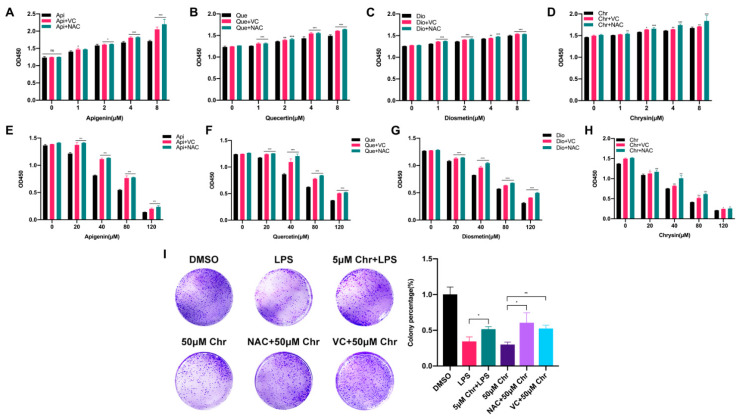
The dual effects of flavonoids on the proliferation of MCF-7 breast cancer cells depending on different drug doses. (**A**) The cell number of MCF-7 cells pretreated with NAC or VC for 6 h and thereafter receiving the indicated dose of apigenin for 60 h. (**B**) The cell number of MCF-7 cells pretreated with NAC or VC for 6 h and thereafter receiving the indicated dose of quercetin for 60 h. (**C**) The cell number of MCF-7 cells pretreated with NAC or VC for 6 h and thereafter receiving the indicated dose of diosmetin for 60 h. (**D**) The cell number of MCF-7 cells pretreated with NAC or VC for 6 h and thereafter receiving the indicated dose of chrysin for 60 h. (**E**–**H**) The cell number of MCF-7 cells pretreated with NAC or VC for 6 h and thereafter receiving the indicated high dose of four flavonoids for 48 h, respectively. (**I**) (**Left**) Colony formation assay of MCF-7 cells treated with 5 μg/mL LPS and/or indicated dose of chrysin. LPS: cells were treated with 5 μg/mL LPS for 48 h, then provided with fresh medium for one week; 5 μM Chr + LPS: cells were pretreated with 5 μM chrysin for 6 h and then 5 μg/mL LPS for 48 h, then provided with fresh medium for one week; 50 μM Chr: cells were treated with 50 μM chrysin for 48 h, then provided with fresh medium for one week; NAC + 50 μM Chr: cells were pretreated with 5 μM NAC for 6 h and then 50 μM chrysin for 48 h, then provided with fresh medium for one week; VC + 50 μM Chr: cells were pretreated with 500 μM VC for 6 h and then 50 μM chrysin for 48 h, then provided with fresh medium for one week. (**Right**) Quantitative analysis. Data was shown as the mean ± SD. * *p* < 0.05, ** *p* < 0.01, *** *p* < 0.001. ns indicates no significant difference.

To explore the correlation between flavonoid doses and cell proliferation, four flavonoids with a series of low or high doses were used to treat MCF-7 breast cancer cells. We found that a low dose of flavonoids ranging from 1 to 8 μM for 60 h slightly enhanced the cell growth, especially at 8 μM, and some of them started to work at 1 μM. Besides, the pretreatment with VC or NAC for 6 h synergistically promoted the cell growth, in line with previous results (Figure 2A–D). Conversely, a high dose of flavonoids ranging from 20 to 120 μM for 48 h markedly inhibited cell proliferation and induced cell death due to the excessive oxidative stress since it could be partly rescued by VC or NAC pretreatment (Figure 2E–H). Colony formation assay was also applied to evaluate the colonization ability of cancer cells. The results showed that pretreatment with 5 μM chrysin markedly restored the LPS or Rosup inhibited colony numbers (Figure 2I and Figure S3C). However, among high-dose groups, 50 μM chrysin significantly downregulated the colony numbers, and the NAC or VC supplement attenuated such suppression. Taken together, these results showed that flavonoids had dual effects on cancer cells depending on the drug doses and treating time, which was relevant to oxidative stress.

### 3.3. Dual Effects of Flavonoids on Physiological Redox Biomarkers and Relevant Gene Expression Levels

**Figure 3:**
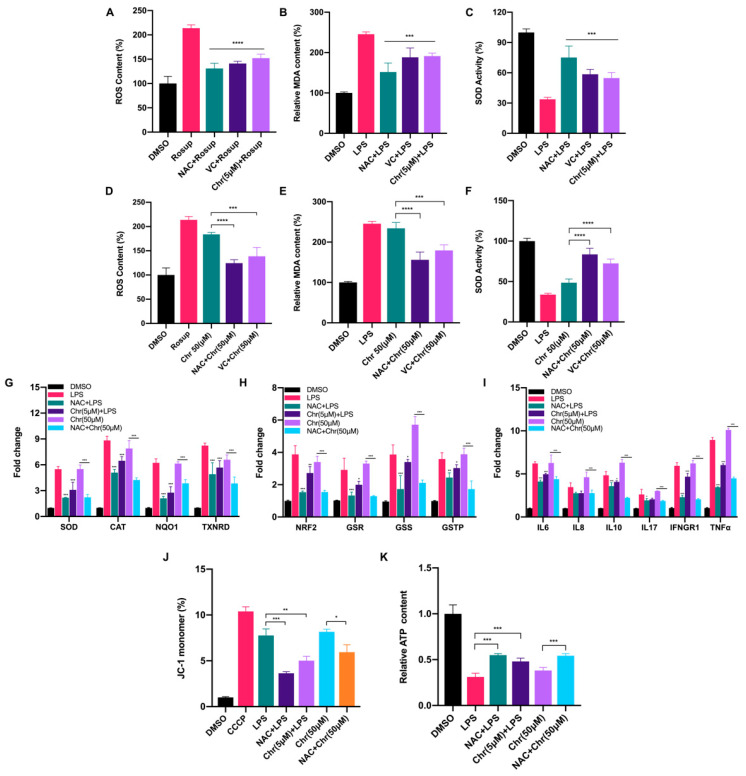
Dual effects of flavonoids on cellular redox biomarkers and relevant gene expression levels. (**A**) The ROS content of different groups. Rosup: 50 μg/mL for 24 h. NAC + Rosup: pretreated with 5 μM NAC for 6 h and then 50 μg/mL Rosup for 18 h. VC + Rosup: pretreated with 500 μM VC for 6 h and then 50 μg/mL Rosup for 18 h. Chr (5 μM) + Rosup: pretreated with 5 μM chrysin for 6 h and then 50 μg/mL Rosup for 18 h. (**B**) The MDA content of different groups. LPS: 5 μg/mL for 24 h. NAC + LPS: pretreated with 5 μM NAC for 6 h and then 5 μg/mL LPS for 18 h. VC + LPS: pretreated with 500 μM VC for 6 h and then 5 μg/mL LPS for 18 h. Chr (5 μM) + LPS: pretreated with 5 μM chrysin for 6 h and then 5 μg/mL LPS for 18 h. (**C**) The SOD activity of different groups. The treatment was similar to (**B**). (**D**) The ROS content of different groups. Rosup: 50 μg/mL for 24 h. Chr (50 μM): 50 μM chrysin for 24 h. NAC + Chr (50 μM): pretreated with 5 μM NAC for 6 h and then 50 μM chrysin for 18 h. VC + Chr (50 μM): pretreated with 500 μM VC for 6 h and then 50 μM chrysin for 18 h. (**E**) The MDA content of different groups. LPS: 5 μg/mL for 24 h. NAC + Chr (50 μM): pretreated with 5 μM NAC for 6 h and then 50 μM chrysin for 18 h. VC + Chr (50 μM): pretreated with 500 μM VC for 6 h and then 50 μM chrysin for 18 h. (**F**) The SOD activity of different groups. The treatment was similar to (**E**). (**G**,**H**) The qPCR results of antioxidative genes. Chr (5 μM) + LPS: pretreated with 5 μM chrysin for 6 h and then 5 μg/mL LPS for 18 h. Chr (50 μM): 50 μM chrysin for 24 h. NAC + Chr (50 μM): pretreated with 5 μM NAC for 6 h and then 50 μM chrysin for 18 h. (**I**) The qPCR results of inflammatory genes. (**J**) The percentage of JC-1 monomers in different groups. Higher proportion of JC-1 indicates more grievous mitochondrial membrane potential collapse. Cells were treated the same as described above. (**K**) The relative ATP content of different groups. Cells were treated the same as described above. Data was shown as the mean ± SD. * *p* < 0.05, ** *p* < 0.01, *** *p* < 0.001, **** *p* < 0.0001. ns indicates no significant difference.

To elucidate the role of flavonoids involved in the oxidative response, we measured the content of ROS, MDA and SOD activity in MCF-7 breast cancer cells. In general, a low dose of chrysin (5 μM) significantly alleviated the oxidative stress induced by LPS or Rosup, characterized by reduced ROS and MDA content but increased SOD activity, performing like reductants NAC/VC (Figure 3A–C). However, a high dose of chrysin (50 μM) showed the effect of promoting oxidative stress, which can be partially reversed by reductants (Figure 3D–F). We also detected the fluorescence intensity of ROS by flow cytometry (Figures S1B and S2A) under different doses of chrysin. Next, the expression levels of antioxidant and inflammatory genes were detected (Figure 3G–I). LPS for 24 h significantly increased the expression of antioxidant genes such as SOD and NAD(P)H quinone dehydrogenase 1 (NQO1), indicating the perturbed redox status. NAC pretreatment alleviated such oxidative stress and restored the imbalance to some extent. Similarly, low-dose chrysin also attenuated the expression of antioxidant genes stimulated by LPS, suggesting its intracellular antioxidant function; however, high-dose chrysin directly activated the expression of these genes, indicating the disorder of cellular redox state. Oxidative stress is usually accompanied by a rapid inflammatory response. We also observed a similar effect on the expression level of inflammatory genes, especially cytokines such as IL-6, IFN-γ and TNF-α, which was examined in macrophage RAW 264.7 cells for further verification (Figure S3A). To further explore which type of ROS was increased following treatment with high-dose flavonoids, MitoSOX Red and CellROX Deep Red probes were used for detection. MitoSOX Red indicated that mitochondria superoxide around the nucleus was significantly enhanced after 50 μM chrysin treatment for 24 h, illustrating that a high dose of flavonoids widely induced oxidative stress both in mitochondria and cytoplasm (Figure S1A). Moreover, the fluorescence intensity of CellROX Deep Red demonstrated that high-dose apigenin triggered strong oxidative stress in the cytoplasm, in line with the above results (Figure S1C). Considering the accumulation of mitochondrial superoxide, we next detected mitochondrial membrane potential (MMP) and ATP production. LPS and 50 μM chrysin induced a higher percentage of JC-1 monomers, indicating an impaired mitochondria function (Figure 3J). Not surprisingly, ATP synthesis was inhibited after high-dose chrysin treatment (Figure 3K). NAC or low-dose chrysin alleviated such energy inhibition, consistent with JC-1 monomers. Taken together, these results presented that flavonoids have a dual effect on the redox of cancer cells, depending on drug doses.

### 3.4. High Dose of Apigenin or Quercetin Induced Cell Apoptosis and Cell Cycle Arrest to Restrain the Proliferation of MCF-7 Breast Cancer Cells

**Figure 4:**
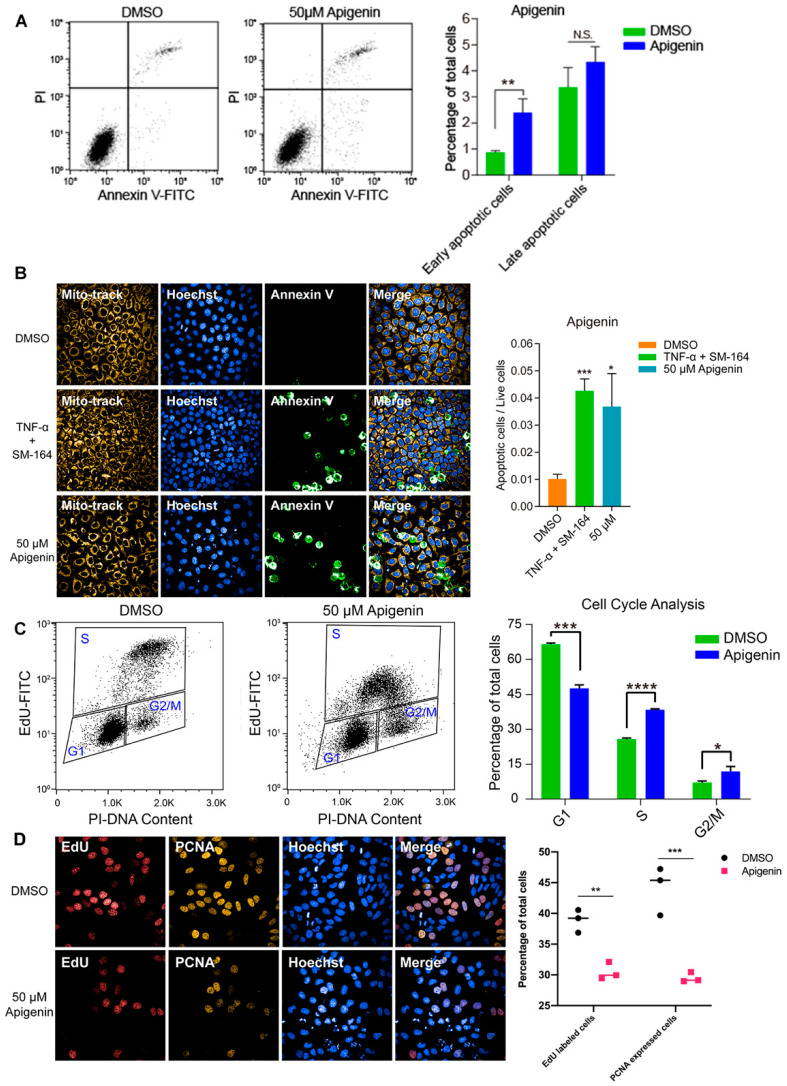
High dose of apigenin induced apoptosis and cell cycle arrest in MCF-7 cells. (**A**) Flow cytometry and statistical results of apoptosis in MCF-7 cells treated with DMSO or 50 μM apigenin for 48 h. TNF-α+SM-164 was positive control. (**B**) Immunofluorescence staining of annexin V and statistical results of apoptosis in MCF-7 cells treated with DMSO or 50 μM apigenin for 48 h. (**C**) Flow cytometry and statistical results of cell cycle arrest in MCF-7 cells treated with DMSO or 50 μM apigenin for 48 h. (**D**) Immunofluorescence results of indicators EdU and PCNA in MCF-7 cells treated with DMSO or 50 μM apigenin for 48 h. Cells were observed under 40× oil laser confocal microscope. Data was shown as the mean ± SD. * *p* < 0.05, ** *p* < 0.01, *** *p* < 0.001, **** *p* < 0.0001. ns indicates no significant difference.

**Figure 5:**
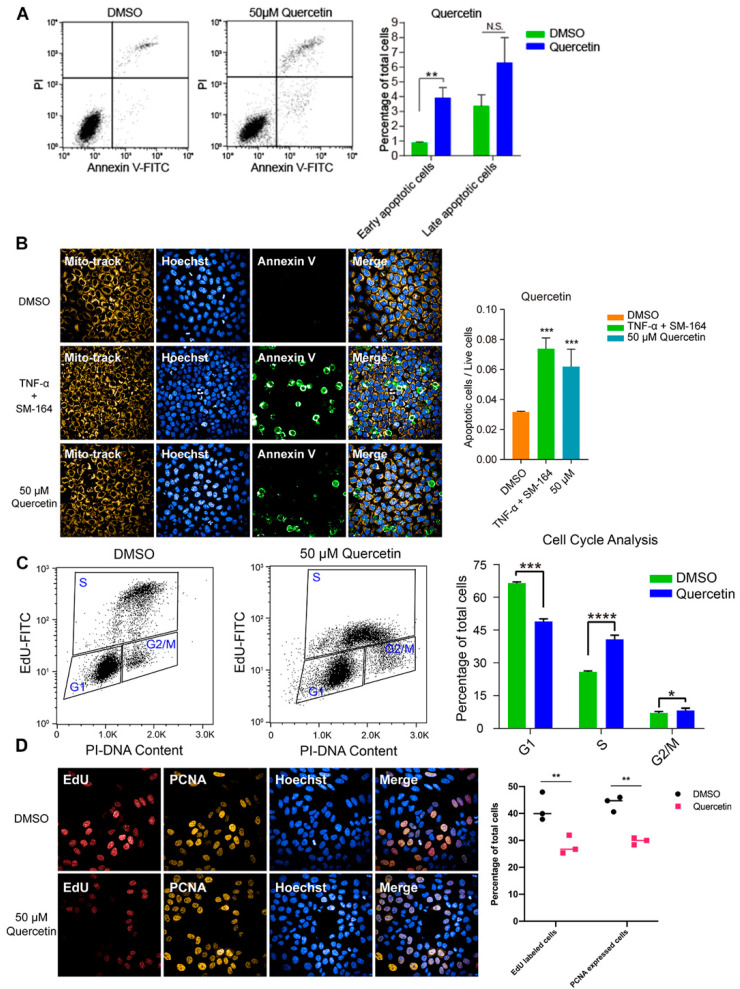
High dose of quercetin induced apoptosis and cell cycle arrest in MCF-7 cells. (**A**) Flow cytometry and statistical results of apoptosis in MCF-7 cells treated with DMSO or 50 μM quercetin for 48 h. TNF-α+SM-164 was positive control. (**B**) Immunofluorescence staining and statistical results of apoptosis in MCF-7 cells treated with DMSO or 50 μM quercetin for 48 h. (**C**) Flow cytometry and statistical results of cell cycle arrest in MCF-7 cells treated with DMSO or 50 μM quercetin for 48 h. (**D**) Immunofluorescence results of indicators EdU and PCNA in MCF-7 cells treated with DMSO or 50 μM quercetin for 48 h. Cells were observed under 40× oil laser confocal microscope. Data was shown as the mean ± SD. * *p* < 0.05, ** *p* < 0.01, *** *p* < 0.001, **** *p* < 0.0001. ns indicates no significant difference.

To investigate the inhibitory effect of high-dose flavonoids on the growth of breast cancer cells, 50 μM apigenin and quercetin were used to treat MCF-7 cells for 48 h. As shown in Figure 4A, the proportions of early apoptosis and late apoptosis in the DMSO treatment group were 0.87% and 3.37%; the proportions of early apoptosis and late apoptosis in the apigenin treatment group were 2.40% and 4.34%, respectively, increasing by 1.53% and 0.97%, showing that 50 μM apigenin induced mild apoptosis in the early stage. Annexin V, being the biomarker of apoptosis, was applied for staining to observe the apoptosis. Here, TNF-α was used as a positive control to induce apoptosis. Obviously, 50 μM apigenin was able to enhance the fluorescence intensity of annexin V, indicating the surge of apoptosis (Figure 4B). In terms of the cell cycle, 50 μM apigenin enhanced the cell distribution of both S and G2/M phases and markedly decreased the proportion of the G1 phase (Figure 4C). To intuitively observe the changes, immunofluorescence was confirmed via EdU and PCNA (proliferating cell nuclear antigen) staining. EdU (5-ethynyl-2′-deoxyuridine) is based on the incorporation of a thymidine analog in the process of DNA synthesis. PCNA is an auxiliary protein of DNA polymerase delta and is involved in the control of eukaryotic DNA replication by increasing the polymerase’s processibility during elongation of the leading strand. Both biomarkers reflect the speed of cell replication and proliferation. Compared with control, the amounts of EdU- and PCNA -labeled cells treated with 50 μM apigenin were markedly decreased, presenting the inhibition of cell replication (Figure 4D). Overall, the proliferation inhibition induced by a high concentration of apigenin mainly resulted from cell cycle arrest, since the percentage of induced apoptosis was relatively low. Similarly, 50 μM quercetin triggered apoptosis and cell cycle arrest as well, leading to the same proliferative inhibition as apigenin (Figure 5).

### 3.5. The Transcriptome Analysis of High Dose of Apigenin or Quercetin in MCF-7 Breast Cancer Cells

**Figure 6:**
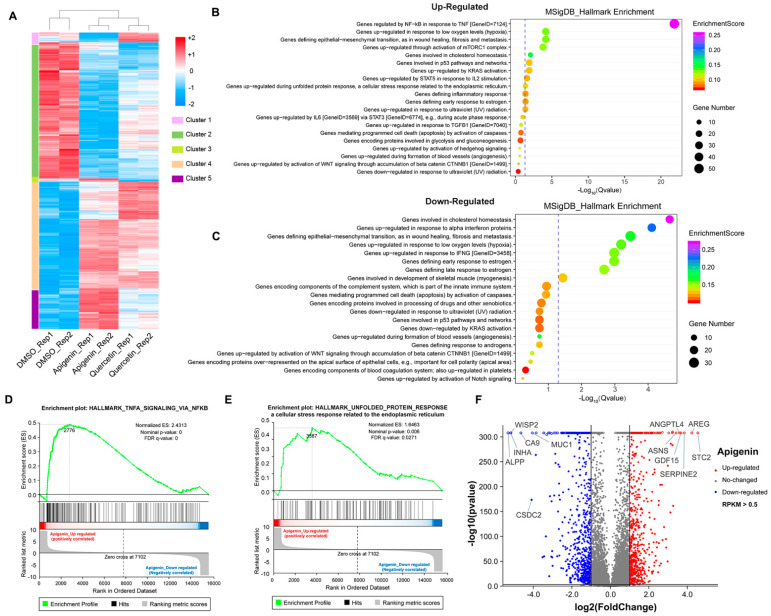
Transcriptome analysis of MCF–7 cells treated with high dose of flavonoids. (**A**) The heat map of cells treated with DMSO or 50 μM apigenin/quercetin for 48 h. (**B**) The GO analysis of cells treated with 50 μM apigenin for 48 h (upregulated). (**C**) The GO analysis of cells treated with 50 μM apigenin for 48 h (downregulated). (**D**) The GSEA analysis of cells treated with 50 μM apigenin for 48 h. TNF-α signaling was enriched and upregulated. (**E**) The GSEA analysis of cells treated with 50 μM apigenin for 48 h. Unfolded protein response signaling was enriched and upregulated. (**F**) The volcano plot revealing the most differentially expressed genes of cells treated with 50 μM apigenin for 48 h. The selection of differentially expressed genes from the transcriptome was based on log_2_|fold change| > 1, *p* value < 0.01.

**Figure 7:**
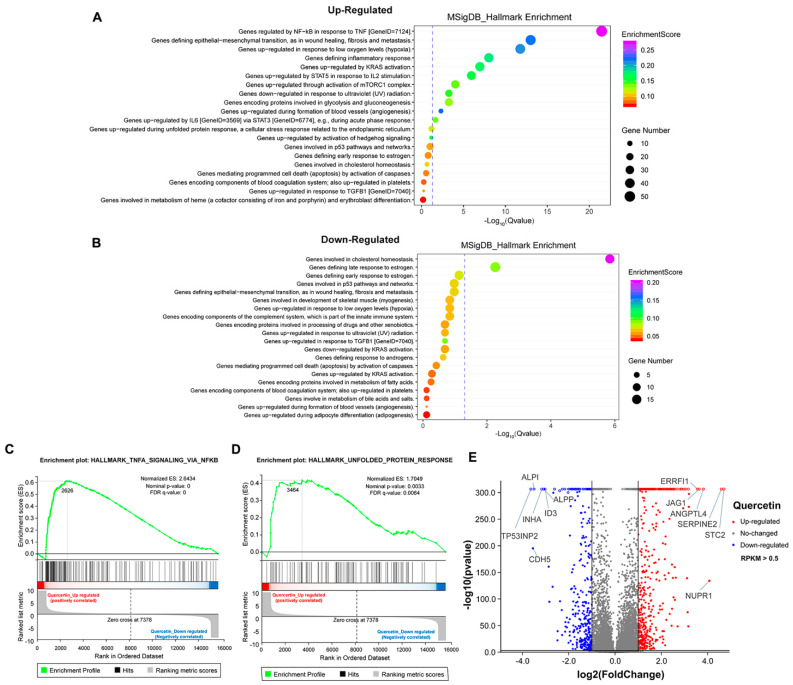
Transcriptome analysis of MCF–7 cells treated with high dose of quercetin. (**A**) The GO analysis of cells treated with 50 μM quercetin for 48 h (upregulated). (**B**) The GO analysis of cells treated with 50 μM quercetin for 48 h (downregulated). (**C**) The GSEA analysis of cells treated with 50 μM quercetin for 48 h. TNF-α signaling was enriched and upregulated. (**D**) The GSEA analysis of cells treated with 50 μM quercetin for 48 h. Unfolded protein response signaling was enriched and upregulated. (**E**) The volcano plot revealing the most differentially expressed genes of cells treated with 50 μM quercetin for 48 h. The selection of differentially expressed genes from the transcriptome was based on log_2_|fold change| > 1, *p* value < 0.01.

To further explore the mechanism of growth inhibition in MCF-7 breast cancer cells, we systematically compared the transcriptomes between the DMSO- and flavonoid-treated groups (apigenin and quercetin, 50 μM for 48 h). The heat map revealed five distinct clusters differing between DMSO- and flavonoid-treated cells, suggesting that apigenin and quercetin resulted in significant alterations of gene expression patterns (Figure 6A). Importantly, the results of GO analysis presented that a high dose of apigenin contributed to the enhanced expression of genes regulated by TNF-α signaling, further supporting the previous results of inflammation response (Figure 6B). We also noticed that apigenin and quercetin inhibited the expression of genes correlated with cholesterol homeostasis, suggesting that apigenin may be able to interrupt the biosynthesis or metabolism of cholesterol in cancer cells (Figure 6C and Figure 7B). Moreover, the GSEA plots presented that TNF-α and unfolded protein response signaling pathways, both reflecting the activation of oxidative stress, were markedly upregulated after apigenin treatment (Figure 6D,E). Besides, multiple genes specifically regulated by apigenin at the transcriptional level were demonstrated in the volcano plot (Figure 6F). Interestingly, quercetin showed similar results to those of apigenin in GO pathways, in which TNF-α signaling and unfolded protein response were largely stimulated in quercetin-treated cells (Figure 7A–D). Finally, the volcano plot of most altered genes was summarized (Figure 7E). Taken together, the transcriptome analysis of apigenin and quercetin displayed highly similar patterns, and both patterns revealed that high doses of flavonoids triggered the activation of oxidative stress and inflammation reaction.

### 3.6. BRCA1 Alleviated the Oxidative Stress and Inflammation Induced by High Dose of Flavonoids

**Figure 8:**
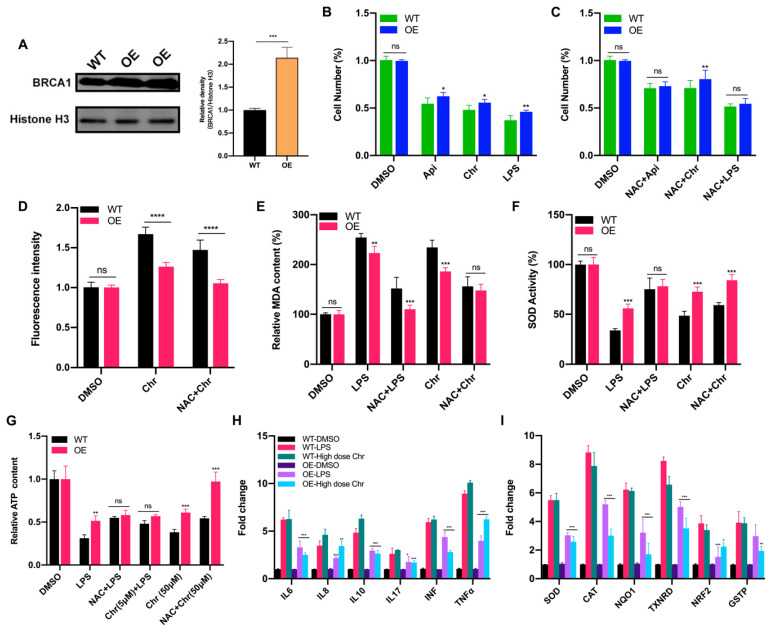
BRCA1 overexpression attenuated the oxidative stress induced by LPS or high dose of flavonoids. (**A**) The Western blot of wild type (WT) and BRCA1-overexpressing cells (OE). (**B**) The cell number of WT/OE cells treated with LPS or 50 μM apigenin/chrysin for 48 h. (**C**) The cell number of WT/OE cells pretreated with 5 μM NAC for 6 h and then 50 μM apigenin/chrysin or 5 μg/mL LPS for 48 h. (**D**) The fluorescence intensity of ROS detected by DCFH-DA assay. Chr: 50 μM chrysin for 24 h. NAC + Chr: pretreated with 5 μM NAC for 6 h and then 50 μM chrysin for 18 h. (**E**) The MDA content of WT/OE cells. LPS: 5 μg/mL LPS for 24 h. NAC + LPS: pretreated with 5 μM NAC for 6 h and then LPS for 18 h. (**F**) The SOD activity of WT/OE cells. (**G**) The relative ATP content of different groups. (**H**) The qPCR results of inflammatory genes in WT/OE cells. LPS: 5 μg/mL LPS for 24 h. High dose Chr: 50 μM chrysin for 24 h. The marked significance of OE groups was obtained by comparing the WT groups with the same treatment, respectively. (**I**) The qPCR results of antioxidative genes in WT/OE cells. The marked significance of OE groups was obtained by comparing the WT groups with the same treatment, respectively. Data was shown as the mean ± SD. * *p* < 0.05, ** *p* < 0.01, *** *p* < 0.001, **** *p* < 0.0001. ns indicates no significant difference.

**Figure 9:**
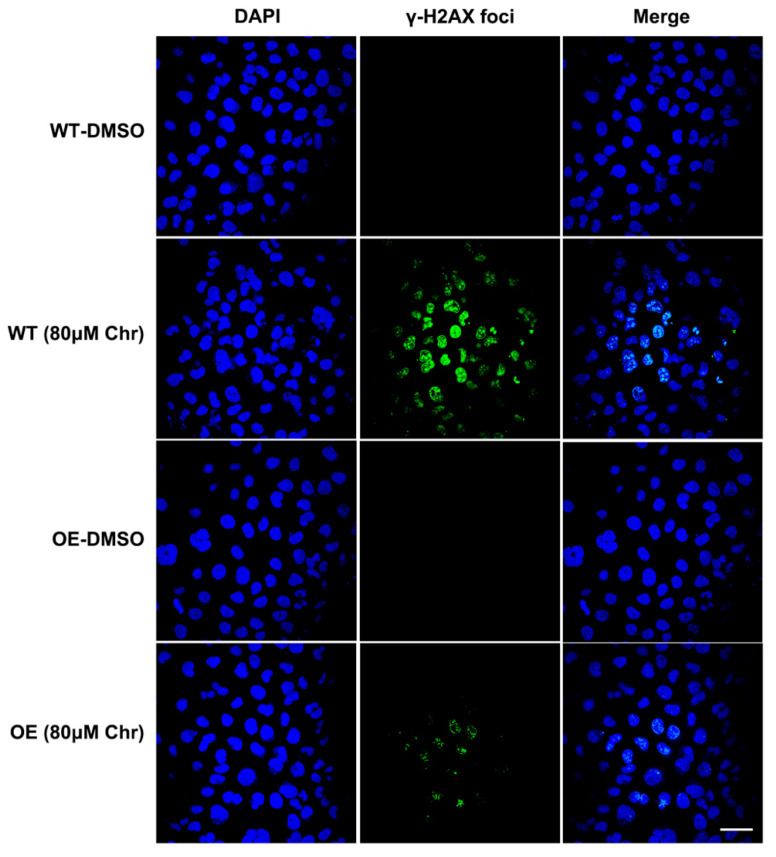
Immunofluorescence results of γH2AX foci. Wild type (WT) or BRCA1-overexpressing cells (OE) were treated with DMSO or high dose of chrysin (80 μM) for 24 h, respectively. DNA damage biomarker γ-H2AX (green) and nuclear DNA stained with DAPI (blue) was presented. Cells were observed under 60× oil laser confocal microscope.

Breast cancer susceptibility gene 1 (BRCA1) is one of the most frequently mutated tumor suppressor genes in human breast cancers. In recent years, BRCA1 has been identified as an essential player in regulating intracellular oxidative stress [28]. Bae et al. found BRCA1 upregulated the expression of multiple genes involved in the cytoprotective antioxidant response, including glutathione S-transferases, oxidoreductases and other antioxidant genes. Meanwhile, BRCA1 overexpression conferred resistance while BRCA1 deficiency conferred sensitivity to oxidizing agents in vitro and in vivo [29,30,31,32]. To investigate whether BRCA1 modulates oxidative stress induced by high-dose flavonoids, BRCA1-overexpressing cells were constructed and verified in MCF-7 cells (Figure 8A). Without drug treatment, no difference in the growth rate of BRCA1-overexpressing cells (OE) and wild-type cells (WT) was observed. The survival rate of OE cells was much higher than that of WT cells under stimulation, indicating that BRCA1 overexpression effectively alleviated such oxidative stress and thereby attenuated cell death (Figure 8B). With the NAC pretreatment, no difference in cell number was observed (Figure 8C). Interestingly, the cell number of OE cells treated with NAC and chrysin was still higher than that of WT, suggesting that NAC did not completely offset the chrysin-induced oxidative stress, which indicated that chrysin may trigger cell death via other non-oxidative-stress pathways. Next, the levels of SOD, MDA and ROS were detected. BRCA1 overexpression significantly rescued the oxidative stress stimulated by LPS or high-dose chrysin, evidenced as decreased ROS and MDA but elevated SOD activity in comparison to WT cells (Figure 8D–F). Nevertheless, such difference disappeared with NAC intervention. Moreover, BRCA1 improved the ATP production inhibited by LPS or 50 μM chrysin that was eliminated by the addition of NAC or 5 μM chrysin (Figure 8G). Finally, the expression level of antioxidant and inflammatory genes further supported the conclusion (Figure 8H,I). For many years, BRCA1 has been well established as a tumor suppressor, and it functions primarily by maintaining genome integrity [33]. We then hypothesized that BRCA1 attenuates the oxidative stress induced by high-dose flavonoids by repairing DNA damage. H2AX phosphorylation at Ser139 (γ-H2AX) is a typical indicator of DNA double-strand breaks after different genotoxic stresses, including ionizing radiation, environmental agents and chemotherapy drugs. The fluorescence intensity of γ-H2AX foci was significantly reduced in OE cells when exposed to a high dose of chrysin compared with WT cells (Figure 9). Taken together, these results presented that BRCA1 effectively resisted the oxidative stress induced by a high dose of flavonoids, possibly by repairing DNA damage. 

## 4. Discussion

Free radicals such as ROS in the human body are gradually accumulated spontaneously under normal conditions and trigger cell death or carcinogenesis depending on a certain level. Here, a low dose of flavonoids slightly stimulated the proliferation of cancer cells under 60 h treatment, but not in short periods such as 24 h. Therefore, we proposed a hypothesis that a low dose of flavonoids possibly eliminated the accumulated free radicals and other oxidants during normal growth, and the subsequent medium exchanging experiments verified this conjecture. Although flavonoids have been identified to display remarkable antioxidant effects in vitro and in vivo, the option of appropriate concentration was still suspicious since a single dose for a certain purpose was mostly used. Besides, extracts of natural products were usually tested rather than single components. The chemicals of extracts are complex and interact with each other, making it difficult to determine the specific biofunction. More importantly, most studies focused on the antioxidant ability of substances in a single dose instead of exploring various concentrations to confirm the possible functional activity at the beginning. Recently, the anti-inflammation and antioxidant activities of natural flavonoids and extracts have been extensively investigated in keratinocytes, melanocytes and fibroblasts when exposed to ultraviolet (UV) [34,35]. Meanwhile, some studies focused on the antioxidant function of flavonoids in relieving chronic oxidative stress in mice with specific diseases of diabetes, drug-induced cancer or polycystic ovary syndrome. Different from the external oxidative stress caused by UV or animal models, our results showed that the oxidation products formed during the normal growth could seriously impede the proliferation of cells as well, aside from nutrition exhaustion. One of the main results we obtained is that a high dose of flavonoids (usually more than IC_50_) can induce severe oxidative stress in cancer cells and increase ROS levels. ROS are important in regulating normal cellular processes and deregulated ROS lead to the development of diseased states in humans, including cancers. Several studies have been found to be marked with increased ROS production which activates protumorigenic signaling, enhances cell survival and proliferation and drives DNA damage and genetic instability [36]. In general, the level of ROS in cancer cells is higher than that in normal cells; this is because tumor cells develop a mechanism of adapting to the high ROS condition by expressing enhanced levels of antioxidant genes while developing neoplastic signaling. However, ROS exceeding a certain threshold can lead to cell disaster and benefits antitumorigenic signaling through initiating oxidative stress, which could be used as a potential target for cancer therapies [37]. Interestingly, the elevated level of ROS induced by high-dose flavonoids was quite sufficient to trigger oxidative stress, thereby promoting cancer cell death. Transcriptome analysis presented that the unfolded protein reaction (UPR) was positively enriched by high doses of apigenin and quercetin. Whether such UPR activation was dependent on enhanced ROS has not yet been determined. Studies have shown that UPR is necessary to maintain the integrity of the endoplasmic reticulum and prevent oxidative stress [38]. Moreover, oxidative stress can induce mitochondrial dysfunction and unfolded protein response in retinal pigment epithelial cells, consistent with our results [39]. However, excessive protein synthesis and unfolded protein accumulation in ER also lead to oxidative stress [40]. In yeasts, UPR and oxidative stress regulate each other, constructing a complicated regulatory network [41,42]. Therefore, the enriched UPR could be the result or cause of oxidative stress.

Previous studies have verified that quercetin and apigenin promoted cell death at high concentrations, and the death mechanisms mainly involve apoptosis, cell cycle arrest and autophagy [43,44,45,46,47,48]. Our results further reveal that a high dose of flavonoids triggered mitochondrial damage and ATP reduction, thus illustrating the cell death from another perspective. Mitochondria are one of the most prominent sources of ROS that contribute to oxidative stress. The electron transport chain located on the inner mitochondrial membrane generates the majority of mitochondrial ROS during the process of oxidative phosphorylation (OXPHOS). Several lines of evidence suggested that oxidative stress as a vital cause or consequence of mitochondrial dysfunction is one of the leading drivers of this cell death program [49]. In addition to producing a large amount of ROS, damaged mitochondria can also be exacerbated by oxidative stress, as seen in Parkinson’s and Alzheimer’s diseases. Aside from the decisive role in modulating endogenous apoptosis, mitochondria are also the main manufactory producing ATP [50]. Mitochondrial damage caused by imbalanced membrane potential directly leads to inhibited energy production and then cell death. NAC or VC pretreatment significantly recovered such effect, further proving that oxidative stress participated in ATP regulation. Indeed, flavonoids (baicalein, luteolin, naringenin and quercetin) were found to suppress the Fenton reaction of the iron–ATP complex to inhibit ATP synthesis [51]. Besides, a study showed that the pro-oxidant activity of flavonoids can contribute to their health-promoting activity by inducing important detoxifying enzymes via a supposed toxic chemical reaction, which was another explanation for the toxicity of high-dose flavonoids [52]. Although the antioxidative properties of flavonoids have been investigated for many years, there are queries about what reactions they will perform in protecting or killing cancer cells. Our findings provide new perspectives for better use of flavonoids as antitumor agents.

Intriguingly, inflammation was markedly activated by high-dose flavonoids, as measured by upregulated TNF-α signaling from transcriptome analysis. TNF-α is a cytokine that binds to TNFRSF1A/TNFR1 and TNFRSF1B/TNFBR, is secreted by macrophages and induces tumor cell death and other inflammatory mediators and proteases that orchestrate inflammatory responses. TNF-α could also be produced by tumor cells and act as an endogenous tumor promoter or suppressor. The role of TNF-α has been linked to all steps involved in tumorigenesis, including transformation, proliferation, invasion, angiogenesis and metastasis [53]. In the past, most flavonoids or natural products were found to exert an inhibitory effect on the inflammatory response via reducing the secretion of cytokines or inhibiting transcription factors such as NF-kB, PPAR, AP-1 and Nrf2 and downstream gene expression [54,55,56,57]. However, our results showed that similar to the oxidative stress induction, a high dose of flavonoids activated the inflammatory response, as supported by transcriptome and qPCR results. To be more accurate, macrophages as a cell model were used for investigation, and upregulated expression levels of the above inflammatory genes were observed. Compared with macrophages, the fold change of the above genes was found lower in MCF-7 cancer cells, indicating that macrophages were more sensitive to the inflammatory response. We speculated that the regulatory network of inflammation in cancer cells could be more refined due to the genetic mutations and evolutionary escape from immune systems. Huang et al. suggested that quercetin and catechin were of benefit for diabetic vascular complications via promoting inflammation in human monocytes and antioxidant abilities against AGE-mediated oxidative stress [58]. Similarly, preincubation with 2 μM β-carotene tended to inhibit the inflammatory reaction, whereas 20 μM β-carotene significantly increased the secretion of proinflammatory mediators [59]. The secretion of inflammatory cytokines or related protein expression was not measured in this research and should be further detected if possible.

Cholesterol homeostasis plays an essential role in proper cellular and systemic functions. The cellular cholesterol level reflects the dynamic balance between biosynthesis, uptake, export and esterification, a process in which cholesterol is converted to neutral cholesteryl esters either for storage in lipid droplets or for secretion as constituents of lipoproteins. Disturbed cholesterol balance underlies not only cardiovascular disease but also an increasing number of other diseases such as cancers [60]. Commonly, the roles played by cholesterol in cancer development and the potential of therapeutically targeting cholesterol homeostasis is a controversial area in the cancer community. Several epidemiologic studies reported an association between cancer and serum cholesterol levels or statin use, while others suggest that there is no such association. In melanoma, an analysis demonstrated that enhanced expression of cholesterol synthesis genes was associated with decreased patient survival [61]. Loss of CYP27A1 has been determined to dysregulate cholesterol homeostasis in prostate cancer; the same was found for the gene ABCA1 [62,63]. Apolipoprotein E gene was shown to regulate aggressive behaviors in prostate cancer cells by deregulating cholesterol homeostasis [64]. Moreover, cholesterol homeostasis directly affected the drug sensitivity to platinum in ovarian cancer [65]. In our results, both apigenin- and quercetin-treated groups have been observed to significantly inhibit the cholesterol homeostasis pathway, indicating that these flavonoids could possibly modulate intracellular cholesterol biosynthesis or metabolism, thus breaking the energy balance to induce cell death. Previously, red grape juice with multiple flavonoids was found to be able to alter cholesterol homeostasis and increase LDL-receptor activity in human cells in vitro [66]. Likewise, the secretion of hepatocyte ApoB was inhibited by two flavonoids, naringenin and hesperetin, via reduced activity and expression of ACAT2 and MTP [67]. Alcohol-free red wine concentrates were more effective than lovastatin for decreasing total cholesterol in vitro and inhibited cholesterol biosynthesis at a transcriptional level [68]. Interestingly, different flavonoids presented selective modulation for liver X receptor (LXR) activity, which has been found to be involved in adjusting cholesterol homeostasis; among these flavonoids, both apigenin and quercetin activated LXR-β [69]. Nevertheless, Deyhim et al. found that cranberry juice increased antioxidant status without affecting cholesterol homeostasis in orchidectomized rats, possibly because a higher dose was required in vivo for regulating cholesterol condition [70]. Although flavonoids showed diverse mechanisms in the cholesterol maintenance in different cell lines, combined with our results, we speculated that the phenotype of inhibiting cholesterol homeostasis promoted the disorder of energy metabolism and cell death of cancer cells, which was conducive to the aggravation of oxidative stress injury. Taken together, these results revealed that high doses of flavonoids are of great potential in regulating intracellular cholesterol homeostasis, which further enriches our conclusion.

Mutations of BRCA1 are found in a high percentage of hereditary breast and ovarian cancers. Women carrying germline mutations in BRCA1 have a 50–80% lifetime risk of developing breast cancer and a 20–40% lifetime risk of developing ovarian cancer [71]. Generally, BRCA1 has been well established as a tumor suppressor and functions primarily by maintaining genome integrity through DNA repair, especially when cells are exposed to oxidative stress. More and more evidence has suggested that BRCA1 regulates oxidative stress and antioxidant signaling transduction [28]. BRCA1 upregulated the expression of multiple genes involved in the cytoprotective antioxidant response, including glutathione S-transferases, oxidoreductases and other antioxidant genes. Besides, BRCA1 deficiency conferred sensitivity to oxidizing agents (hydrogen peroxide and paraquat) [32]. BRCA1 activated p21 expression and then enhanced the transcriptional level of antioxidant genes [72]. It is physically associated with Nrf2, a master transcription factor modulating antioxidant enzymes, to prevent ubiquitin-dependent degradation by KEAP1 and exert an antioxidant effect [73]. BRCA1 was also found to bind and stabilize hypoxia-inducible factor-1α (HIF-1α) and promote tumor survival [74]. Here, our results showed that BRCA1 suppressed the oxidative stress induced by high-dose flavonoids, possibly by repairing DNA damage, as manifested by fewer γ-H2AX foci. Genome stability is key for normal physiological activities, and the main function of BRCA1 is to keep the genome stable. Considering the fact that ROS directly cause DNA damage, it is plausible that BRCA1 functions like this to regulate oxidative stress. Our results supported and further elucidated the possible mechanism from different levels that BRCA1 restrains oxidative stress via repairing DNA double-strand breaks, highlighting a new perspective that a daily diet containing relatively high doses of flavonoids may be more conducive to tumor inhibition for breast cancer patients, especially for those with BRCA1 mutations or silence, thus leading to synthetic lethality, which could be more beneficial in prolonging prognosis.

## Data Availability

The data presented in this study are available the article and supplementary materials.

## Supplementary Materials

The following supporting information can be downloaded at: https://www.mdpi.com/article/10.3390/antiox11040622/s1, Figure S1: (A) The staining of mitochondria superoxide with MitoSOX Red Indicator. Cells were treated with DMSO or 50 μM chrysin for 24 h, stained and observed under a laser confocal microscope. Mitochondria superoxide (red) and nuclear DNA stained with DAPI (blue) was showed. (B) Fluorescence intensity of ROS detected by flow cytometry with DCFH-DA assay. Rosup: 50 μg/mL for 24 h. NAC + Rosup: pretreated with 5 μM NAC for 6 h thereafter 50 μg/mL Rosup for 18 h. Chr (5 μM) + Rosup: pretreated with 5 μM chrysin for 6 h thereafter 50 μg/mL Rosup for 18 h. Chr (50 μM): treated with 50 μM chrysin for 24 h. NAC + Chr (50 μM): pretreated with 5 μM NAC for 6 h thereafter 50 μM chrysin for 18 h. (C) Fluorescence intensity of oxidative stress detected by flow cytometry with CellROX Deep Red probe. TBHP: treated with 250 µM TBHP for 2 h (positive control). NAC + TBHP: pretreated with 1 mM NAC for 1 h thereafter 250 µM TBHP for 2 h. Api: treated with 100 μM apigenin for 2 h. NAC + Api: pretreated with 1 mM NAC for 1 h thereafter 100 μM apigenin for 2 h; Figure S2: The original flow cytometry plots of Figure S1B; Figure S3: (A) The qPCR results of relevant inflammatory genes in RAW 264.7 macrophages treated with four flavonoids, respectively. LPS: 5 μg/mL LPS for 24 h. Low dose means 5 μM and high dose means 50 μM. NAC pretreatment was 6 h thereafter 18 h of flavonoids treatment. (B) The original flow cytometry plots of Figure 8D. (C) The colony formation of Rosup and Chr (5 μM) + Rosup in MCF-7 cells. The treatment was described before. (D) The original WB gels for BRCA1 overexpression. The gels were cropped according to the targeted protein molecular, respectively. Table S1: The qPCR primers of detected genes; Table S2: The information of flavonoids and antibodies; Table S3: The information of used cell lines.
