# Supplementary material for: The Biphasic Effect of Flavonoids on Oxidative Stress and Cell Proliferation in Breast Cancer Cells
Source: Antioxidants (Basel). 2022 Mar 24;11(4):622. doi: 10.3390/antiox11040622 (PMC9032920; doi:10.3390/antiox11040622)
Supplement: Supplementary file 1 [file antioxidants-11-00622-s001.zip › antioxidants-1575431-supplementary.pdf]

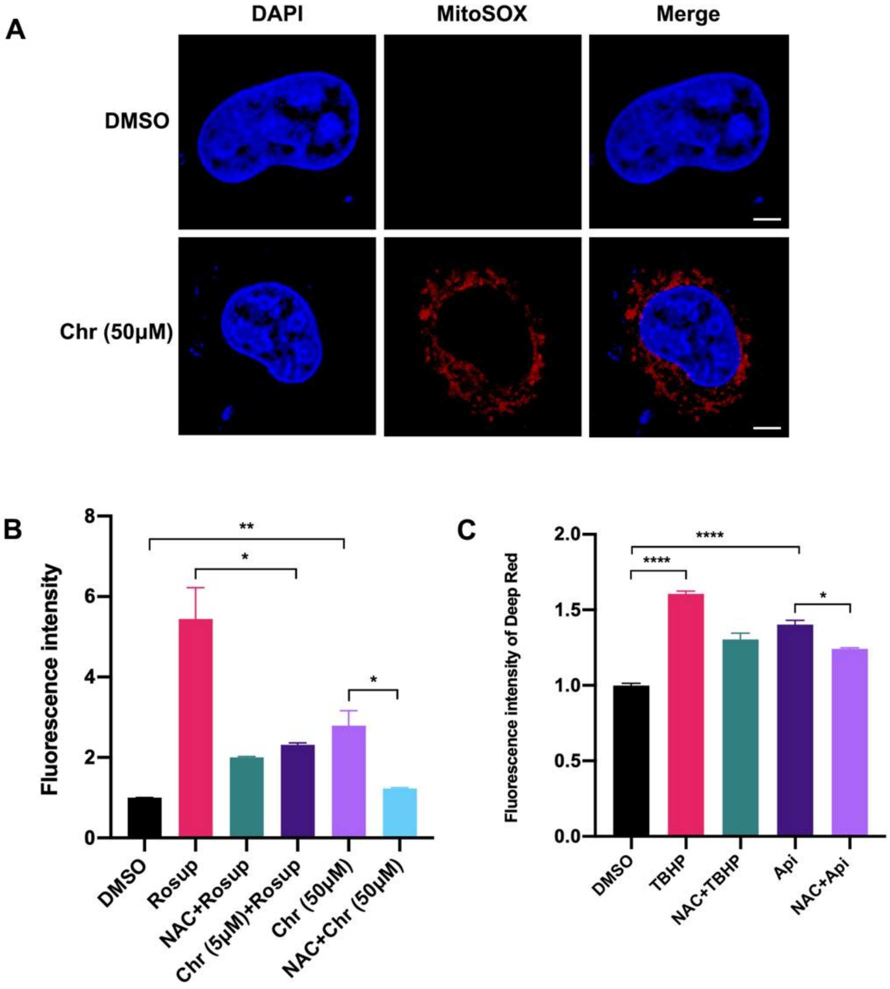

Figure S1: (A) The staining of mitochondria superoxide with MitoSOX Red Indicator. Cells were treated with DMSO or 50 μM chrysin for 24 h, stained and observed under a laser confocal microscope. Mitochondria superoxide (red) and nuclear DNA stained with DAPI (blue) was showed. **(B)** Fluorescence intensity of ROS detected by flow cytometry with DCFH-DA assay. Rosup: 50 μg/ml for 24 h. NAC + Rosup: pretreated with 5 μM NAC for 6 h thereafter 50 μg/ml Rosup for 18 h. Chr (5 μM) + Rosup: pretreated with 5 μM chrysin for 6 h thereafter 50 μg/ml Rosup for 18 h. Chr (50 μM): treated with 50 μM chrysin for 24 h. NAC + Chr (50 μM): pretreated with 5 μM NAC for 6 h thereafter 50 μM chrysin for 18 h. **(C)** Fluorescence intensity of oxidative stress detected by flow cytometry with CellROX Deep Red probe. TBHP: treated with 250 μM TBHP for 2 h (positive control). NAC + TBHP: pretreated with 1 mM NAC for 1 h thereafter 250 μM TBHP for 2 h. Api: treated with 100 μM apigenin for 2 h. NAC + Api: pretreated with 1 mM NAC for 1 h thereafter 100 μM apigenin for 2 h.

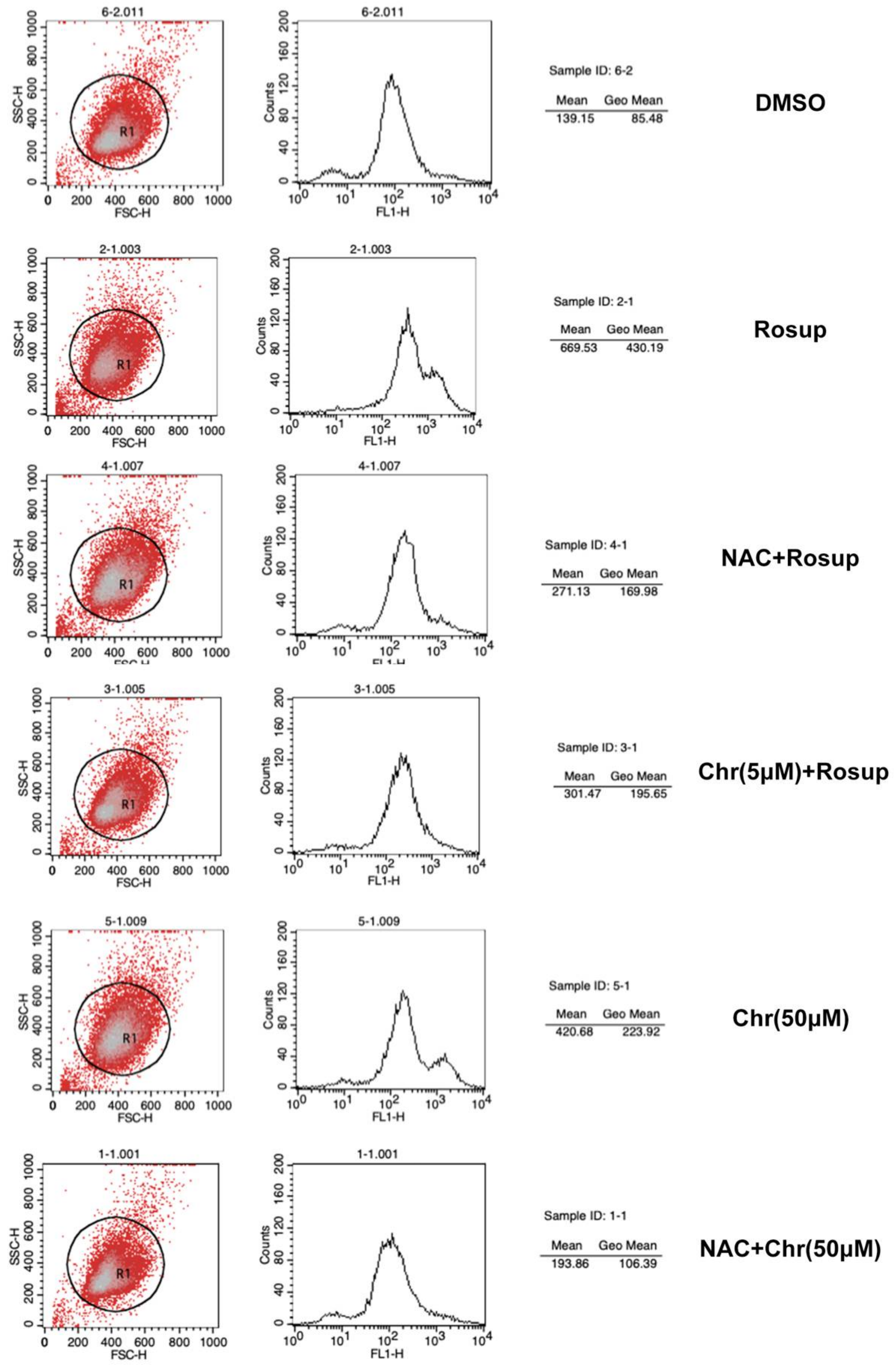

Figure S2 The flow cytometry results of **Figure S1B**.

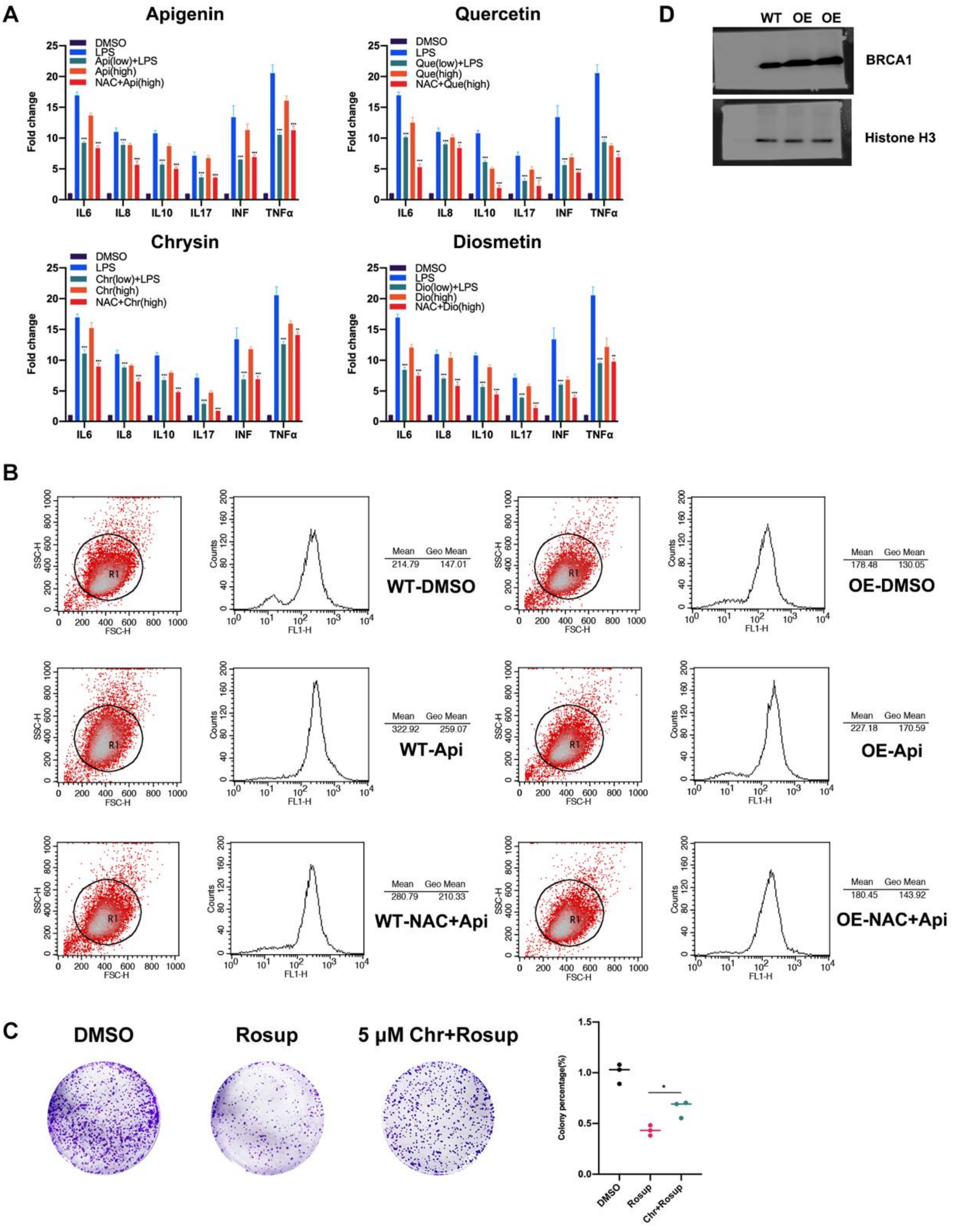

Figure S3: **(A)** The qPCR results of relevant inflammatory genes in RAW 264.7 macrophages treated with four flavonoids, respectively. LPS: 5 μg/ml LPS for 24 h. Low dose means 5 μM and high dose means 50 μM. NAC pretreatment was 6 h thereafter 18 h of flavonoids treatment. **(B)** The original flow cytometry plots of Figure 8D. **(C)** The colony formation of Rosup and Chr (5 μM) + Rosup in MCF-7 cells. The treatment was described before. **(D)** The original WB gels for BRCA1 overexpression. The gels were cropped according to the targeted protein molecular, respectively.

Table S1 The qPCR primers of detected genes

GENE NAME	Accession number	F-primer (5′-3′)	R-primer (5′-3′)
SOD2	NM_000636.4	TGTTGGAGACCTGGGCAATG	CTCTGCCCAAGTCATCTGGTT
CAT	NM_001752.4	TCACTCAGGTGCGGACTTTC	TGGATGCGGGAGCCATATTC
NQO1	NM_000903.3	CAACAGACCAGCCAATCA	ACCTCCCATCCTTTCCTC
GPX1	NM_000581.4	CTTCAACCTGTCCTCCCT	GGTCATTCATCTGGGTGT
TXNRD1	NM_182729.3	GTGTGAATGTGGGTTGCATACC	TCCTCGACGTTCCACCCATA
NRF2	NM_006164.5	AGGACATGGATTTGATT	TACCTGGGAGTAGTTGGCA
GSR	NM_000637.5	CGCTGAGAACCCAGAG	AAACGGAAAGTGGGAACAGTAAGTA
GSS	NM_000178.4	CGAGTGATCCAATGCAT	ATGTCCCACGTGCTTGTTCAT
GSTP1	NM_000852.4	TTTGCGGACTACAACCTG	CCCTCACTGTTTCCCATT
GAPDH	NM_002046.7	GGGTCATCATCTCTGCACCT	GGTCATAAGTCCCTCCACGA
ACTB	NM_001101.5	GTCTCCTCTGACTTCAACAGCG	ACCACCCTGTTGCTGTAGCCAA
IL6	NM_000600.5	GGAGGAAAAGGACGGATGCT	GGTCAGTGTTTGTGGCTGGA
IL8	NM_000584.4	CCTCTTGTTCAATATGACTTCCA	GGCCCACTCTCAATAACTCTC
IL10	NM_000572.3	AGCACTACTCTGTTGCCTGG	TTGGGGTAGACTTTGGGGTCT
IL17	NM_002190.3	AGATTACTACAACCGATCCACCT	GGGGACAGAGTTCATGTGGTA
IFNGR1	NM_000416.3	AGCAGGAAGTCGATTATGATCCC	CTGGCACTGAATCTCGTCACA
TNF-α	NM_000594.4	CCTCTCTCTAATCAGCCCTCTG	GAGGACCTGGGAGTAGATGAG

Table S2 The information of flavonoids and antibodies

NAME	SOURCE	Cat No.
Apigenin	CHENGDU MUST, Chengdu, China	A0113
Chrysin	CHENGDU MUST, Chengdu, China	A0292
Diosmetin	CHENGDU MUST, Chengdu, China	A0927
Quercetin	CHENGDU MUST, Chengdu, China	A0083
Histone H3	Beyotime, Shanghai, China	AF0009
γ-H2AX	Beyotime, Shanghai, China	AF5836
Hoechst 33342	Beyotime, Shanghai, China	C1022
BRCA1	Beyotime, Shanghai, China	AF6339
HRP-labeled Goat Anti-Rabbit IgG	Beyotime, Shanghai, China	A0208
HRP-labeled Goat Anti-Mouse IgG	Beyotime, Shanghai, China	A0216

Table S3 The information of used cell lines

CELL LINE	SPECIES	TYPE	SOURCE
MCF-7	*Homo sapiens*	Breast cancer	ATCC
MDA-MB-231	*Homo sapiens*	Breast cancer	ATCC
4T1	*Mus musculus*	Breast cancer	ATCC
RAW 264.7	*Mus musculus*	Macrophage	ATCC
